# Reduction of bioavailability and phytotoxicity effect of cadmium in soil by microbial-induced carbonate precipitation using metabolites of ureolytic bacterium *Ochrobactrum* sp. POC9

**DOI:** 10.3389/fpls.2023.1109467

**Published:** 2023-06-21

**Authors:** Marta Zakrzewska, Grzegorz Rzepa, Marcin Musialowski, Aleksandra Goszcz, Robert Stasiuk, Klaudia Debiec-Andrzejewska

**Affiliations:** ^1^ Department of Environmental Microbiology and Biotechnology, Institute of Microbiology, Faculty of Biology, University of Warsaw, Warsaw, Poland; ^2^ Department of Mineralogy, Petrography and Geochemistry, Faculty of Geology, Geophysics and Environmental Protection, AGH University of Science and Technology, Krakow, Poland; ^3^ Department of Geomicrobiology, Institute of Microbiology, Faculty of Biology, University of Warsaw, Warsaw, Poland; ^4^ Department of Ecotoxicology, Institute of Environmental Biology, Faculty of Biology, University of Warsaw, Warsaw, Poland

**Keywords:** heavy metals immobilization, ureolytic bacteria, crop plants protection, plants growth promoting bacteria, agricultural soil treatment, remediation in situ

## Abstract

The application of ureolytic bacteria for bioremediation of soil contaminated with heavy metals, including cadmium (Cd), allows for the efficient immobilization of heavy metals by precipitation or coprecipitation with carbonates. Microbially-induced carbonate precipitation process may be useful also in the case of the cultivation of crop plants in various agricultural soils with trace but legally permissible Cd concentrations, which may be still uptaken by plants. This study aimed to investigate the influence of soil supplementation with metabolites containing carbonates (MCC) produced by the ureolytic bacterium *Ochrobactrum* sp. POC9 on the Cd mobility in the soil as well as on the Cd uptake efficiency and general condition of crop plants (*Petroselinum crispum)*. In the frame of the conducted studies (i) carbonate productivity of the POC9 strain, (ii) the efficiency of Cd immobilization in soil supplemented with MCC, (iii) crystallization of cadmium carbonate in the soil enriched with MCC, (iv) the effect of MCC on the physico-chemical and microbiological properties of soil, and (v) the effect of changes in soil properties on the morphology, growth rate, and Cd-uptake efficiency of crop plants were investigated. The experiments were conducted in soil contaminated with a low concentration of Cd to simulate the natural environmental conditions. Soil supplementation with MCC significantly reduced the bioavailability of Cd in soil with regard to control variants by about 27-65% (depending on the volume of MCC) and reduced the Cd uptake by plants by about 86% and 74% in shoots and roots, respectively. Furthermore, due to the decrease in soil toxicity and improvement of soil nutrition with other metabolites produced during the urea degradation (MCC), some microbiological properties of soil (quantity and activity of soil microorganisms), as well as the general condition of plants, were also significantly improved. Soil supplementation with MCC enabled efficient Cd stabilization and significantly reduced its toxicity for soil microbiota and plants. Thus, MCC produced by POC9 strain may be used not only as an effective Cd immobilizer in soil but also as a microbe and plant stimulators.

## 1 Introduction

Cadmium (Cd) is considered a highly toxic and hazardous element due to its damaging properties on proteins’ and nucleic acids’ structure (Lee et al., 2012; Balali-Mood et al., 2021), high bioavailability (Godt et al., 2006; Tran and Popova, 2013) and cancerogenic properties (Lunn et al., 2022). The main routes of human exposure to Cd are smoking and consuming certain crop plants grown in Cd-contaminated soil (Satarug and Moore, 2004; Chaney, 2012; European Food Safety Authority, 2012). The elevated concentration of Cd is more and more frequently observed in agricultural soil (Tran and Popova, 2013) as a result of anthropological activities, e.g. using of sludge and phosphate fertilizers (e.g. superphosphates), which may contain up to 10 mg/kg Cd (Satarug et al., 2003).

According to the current guidelines of the United Nations Economic Commission for Europe, the total concentration of Cd in agricultural soil should not exceed 0.9 mg/kg (de Vries et al., 2003) but the maximum permissible concentration (MPC) for Cd in soil, crop plants and other food types should be set at the lowest possible level (Satarug et al., 2017). Even at trace concentrations, plants can successfully uptake Cd from the soil, often without any visible phytotoxic effects (Benavides et al., 2005). Cadmium phytoextraction rate may be increased by certain environmental conditions, e.g. long periods of drought (Tudoreanu and Phillips, 2004; Henry et al., 2007; Bashir et al., 2019). Drought stress triggers plant roots’ exudation of compounds that not only secure the soil against further water loss (Williams and de Vries, 2020), but also enhance nutrition (Kohli et al., 2012). Secretion of organic acids (e.g. citric, malic, tartaric, oxalic, phenolic) causes local soil acidification, increasing mobility not only of nutrients but also hazardous elements, e.g. Cd, which are more effectively absorbed by plants (Fageria et al., 2002; Tudoreanu and Phillips, 2004; Bashir et al., 2019).

The commonly known remediation methods dedicated to soils contaminated with Cd are often unsuitable for agricultural soils for many reasons (Megharaj and Naidu, 2017) since they significantly change soil physical, chemical, and (micro)biological quality (Dhaliwal et al., 2020). While in the case of post-industrial soils, it is not of great importance, in the case of agricultural soils, changing even one of the above-mentioned parameters may exclude the soil from agricultural use for many years (Dhaliwal et al., 2020). Thus, the treatment of Cd-contaminated agricultural soil should be provided with the use of an effective, highly selective, and environmentally friendly remediation method that does not change the natural soil properties (Hou et al., 2020). An additional benefit of the method could be the improvement of plant nutrition and microbiological fitness of the soil.

Soil bioconsolidation based on microbial-induced carbonate precipitation (MICP) leads to the immobilization of heavy metals, including Cd, in the form of carbonate salts (Liu et al., 2021). MICP is based on the microbiological urea hydrolysis reaction (through the urea decomposition produces ammonia and carbonate ions) and may contribute to the reduction of bioavailable forms of heavy metals in soil (Wang et al., 2019a). MICP is generally regarded as an efficient, environmentally safe, and highly selective method (Rajasekar et al., 2021) and thus it seems to be very promising in the remediation of agricultural soil. Furthermore, in contrast to other remediation methods e.g. surface capping (Li et al., 2019), encapsulation (Khan et al., 2004), or soil-washing (Hashim et al., 2011), MICP may be used for soil treatment even in trace concentrations of contaminants. The high effectiveness of the MICP process has been already verified in the efficient immobilization of copper, arsenic, cadmium, lead and zinc from contaminated soils (Achal et al., 2011; Achal et al., 2012a; Achal et al., 2012b; Kumari et al., 2014; Liu et al., 2021).

The MICP process may contribute not only to the immobilization of Cd and other heavy metals in soil (Kang et al., 2014b) but also, by the use of an appropriate bacterial strain and a proper methodology of soil supplementation, may potentially also stimulate soil microbiota and improve the nutrition of crop plants. Among the metabolites co-produced during the urea decomposition (and simultaneous carbonates production) is not only ammonia (Liu et al., 2021), which is the most easily digestible source of nitrogen for soil microorganisms and plants (Robertson and Groffman, 2015), but also various types of secondary metabolites, such as organic acids, ligands, alcohols, short-chain peptides, and unsaturated fatty acids (Yang et al., 2021) that may enrich the agricultural soil with additional nutrients. Thus, the MICP method with the use of MCC may have a potentially triple beneficial effect on plants growth and development, not only by reducing Cd toxicity and additional nutrition but also by the improvement of the general condition of soil microbiota, eg. quantity and activity. Due to the controlled production of MCC and its reproducibility, the use of metabolites could be superior to inoculation with urea-degrading microorganisms for bioremediation purposes. Since microbial ureolytic activity in soil is influenced by various factors, the outcome of microorganisms’ introduction is difficult to predict (Zhao et al., 2022).

The main aim of the study was to verify the hypothesis about soil microbiota stimulation and crop plants (root parsley) growth promotion by the utilization of the MICP method based on soil supplementation with MCC. The experiments were performed with the use of model crop plants cultivated in agricultural soil contaminated with low concentrations of Cd, which was supplemented with MCC produced by the ureolytic and metabolically versatile bacterium *Ochrobactrum* sp. POC9, isolated from sewage sludge. The ability of the POC9 strain to the production of various potentially beneficial secondary metabolites has been already described in our previous study (Yang et al., 2021). In this study, detailed research aimed at (i) the optimization of carbonate production by *Ochrobactrum* POC9, (ii) the selection of the method for supplementation of Cd-contaminated soil with POC9 metabolites to obtain effective Cd immobilization, (iii) the confirmation of the presence of cadmium carbonate in soil, and (iv) verification of the impact of soil enrichment with POC9 metabolites on the Cd bioavailability, quantity and activity of soil microbes, general condition of plants and the efficiency of Cd uptake by plants were performed. The presented study was realized in the context of the development of an effective method of remediation and nutrition of soils, contaminated with trace concentration of Cd, useful for agricultural purposes.

## 2 Materials and methods

### 2.1 Characteristics of the *Ochrobactrum* sp. POC9 and optimization of its carbonate productivity

As an efficient carbonate producer, the ureolytic bacterium *Ochrobactrum* sp. POC9 was applied. POC9 strain was isolated from raw sewage sludge samples collected from the wastewater treatment plant “Czajka” (Warsaw, Poland). Genomic and physiological characteristics of the *Ochrobactrum* sp. POC9 in the context of sewage sludge utilization (Poszytek et al., 2018) and enhancing biogas production (Poszytek et al., 2019) were previously described. Furthermore, the possibility of utilizing the ureolytic activity of POC9 for the treatment of acidic leachates (Yang et al., 2020) and stimulating the activity of sulfates-reducing bacteria (Yang et al., 2021) was also verified. In our previous paper, the composition of MCC produced on a medium containing urea was also analyzed. Among identified metabolites (alcohols, organic acids, unsaturated hydrocarbons as well as nitrogen and sulfur-containing organic compounds), no organic compounds with Cd immobilizing properties were found (Yang et al., 2020).

The inoculum of the POC9 strain was used for various experiments described in this paper. It was prepared by routine overnight cultivation of the strain in lysogeny broth (LB) medium at room temperature, to reach the appropriate initial optical density (OD) measured spectrophotometrically at wavelength 600 nm (OD_600nm >_1). Then, the inoculum was used for inoculation of various microbial media containing urea. Bacterial cultures in media with urea were carried out for 72 hours, with shaking 150 rpm, in 100 ml final volume of the medium. Urea-containing media was enriched with sodium chloride (5 g/l), phosphate salt (KH_2_PO_4_ 2 g/l) and peptone (1 g/l) (Christensen, 1946; Yang et al., 2020).

The effect of various physical, chemical and microbiological factors on the carbonate productivity of *Ochrobactrum* sp. POC9 in batch cultures was investigated. The carbonate production efficiency depending on the (i) urea concentration (0.5, 1, 2 and 5%), (ii) pH of the medium (5, 5.5, 6, 6.8 and 7.5), (iii) temperature of the culture (22, 30 and 37 °C) and (iv) initial quantity of microorganisms (optical density - OD_600nm_: 0.01; 0.03; 0.06; 0.08; 0.1) was determined. The pH of the media was adjusted as needed with 0.1M HCl or NaOH. Uninoculated media were used as chemical controls. All batch cultures were performed in triplicates. During the batch culture cultivation, the concentration of carbonate was monitored at the end of the experiments (after 72 hours) using Nanocolor^®^ kits (Macherey-Nagel, Germany) according to the instructions given by the manufacturer. As a result of these experiments various compositions of metabolites containing carbonates (MCC) were obtained.

### 2.2 Physico-chemical and microbiological characteristics of Cd-contaminated soil

Commercially available, potted garden soil (fractions< 2 mm) was used in the experiments. The soil was artificially contaminated with Cd in concentration reflecting the natural, environmental conditions in agricultural soil. An aqueous cadmium chloride solution (CdCl_2_*2H_2_O) was added to the air-dry soil to achieve a final Cd concentration of 2 mg/kg of dry-weight soil. Then, the soil was thoroughly mixed to homogenize its entire volume. The soil was incubated at room temperature under a cover and systematically watered with distilled water to maintain a constant humidity of about 50%. Additionally, to further soil homogenization and maintain humidity, the soil was thoroughly mixed every 3 days. Soil conditioning with Cd was carried out for 30 days.

Basic physico-chemical and microbiological parameters of Cd-contaminated soil were determined. The elemental contents of carbon, nitrogen, and sulfur, the total solids, volatile solids, pH, bulk density and porosity of the soil as well as microbiological parameters in the form of heterotrophic bacteria and fungi quantity and dehydrogenase activity of soil microbes were presented in **Table 1**.

**Table 1:** Physico-chemical and microbiological characterization of soil contaminated with Cd, including percentage concentration of carbon, nitrogen, and sulfur, total solids [TS], volatile solids [VS], pH, bulk density, soil porosity, the quantity of heterotrophic microorganisms and activity of dehydrogenase in soil.

Parameters	Units	Values
C	% [v/w]	55.03
N	% [v/w]	13.00
S	% [v/w]	0.66
TS	% fresh mass	52.60
VS	% total solids	13.91
pH	–	5.30
Bulk density	g/cm^3^	0.48
Soil porosity	%	34.29
Quantity of heterotrophic microorganisms	CFU/ml	1.32*10^8^
Dehydrogenase activity	INTF h^-1^g^-1^	11.581

Elementary CHNS analysis was performed by Elemental Analyzer EA1112 (Thermo Finnigan). Total solids were determined by drying the soil sample at 50°C until constant weight. Volatile solids were determined by the thermal treatment of the dried soil sample at 550°C for 6 hours. pH was measured in water by pH-meter (Mettler Toledo F-20, Switzerland) with a compatible pH electrode (LE438). Bulk density was calculated on the basis of the ratio of solid soil weight to total soil volume. Soil porosity was determined based on the specific density and bulk density of the soil.

The quantity of heterotrophic (facultatively or obligatorily) aerobic and cultivable microorganisms (bacteria and fungi) in the soil was determined using the method of plating soil extracts on plates with unselective LB Agar medium. To prepare soil extracts, 5 g of fresh soil was placed in sterile flasks, and then 45 ml of 0.85% NaCl solution was added. The samples were shaken (150 rpm) overnight at room temperature. Then, serial dilutions from the obtained inoculum were prepared and plated per 100 µl on plates. The dishes were incubated at room temperature for 96 hours and then grown colonies were counted. The results were averaged and presented as values in CFU/ml (as colony-forming units per 1 milliliter). The dehydrogenase activity of soil microbes (bacteria, fungi and others) was determined according to the method described by von Mersi and Schinner (1991). All analyses were performed in triplicates.

### 2.3 Optimization of the soil supplementation with MCC

In the frame of the preliminary studies, before soil supplementation with MCC, the process of cadmium precipitation in solution enriched with MCC was investigated. Detailed experimental methodology and results of obtained precipitates analysis were presented in **Supplementary Materials**.

Optimization of soil supplementation was performed with the use of MCC obtained in conditions selected in the frame of the experiment described in section 2.1. They were as follows: 2% urea, OD_600nm_ 0.06, pH 6.8, temperature 23°C. The obtained mixture of MCC was characterized by pH 9.1, Ec 47.6 mS, and carbonate concentration 12 770 mg/l. 200 g of Cd-contaminated soil was supplemented with MCC in the following volume ratios: 0:1 (soil without MCC supplementation), 1:1, 2:1, 3:1, 7:1, 8:1 and 10:1 w/v. The soils enriched with MCC were incubated under constant humidity (50%) and temperature conditions (22°C). The experiment was conducted for 14 days. Soil samples for physico-chemical and microbiological analysis were collected. In the frame of physico-chemical analysis, pH of the soil and the bioavailability of Cd were determined. pH and microbiological analysis of soil were determined according to the methods described in section 2.2.

### 2.4 Investigation of the presence of cadmium carbonate in soil

Due to the low expected concentration of Cd in the studied soil, it was impossible to apply standard analyzes of the phase composition like X-ray diffractometry. Therefore, in order to establish the forms (speciations) of Cd in the soil, Fourier-transform infrared spectroscopy (FTIR) and scanning electron microscopy (SEM) were chosen instead.

Infrared spectra were collected using Thermo Scientific Nicolet 7600 spectrometer in the range of 400–4000 cm^-1^. The spectra reported are the resultant of 64 scans at the resolution of 1 cm^-1^. Prior to analysis, KBr discs were obtained by homogenizing 200 mg of ground potassium bromide with 2 mg of the sample. Scanning electron microscope (SEM) analyses of powder samples were carried out in low vacuum mode, using a FEI 200 Quanta FEG microscope equipped with an EDS/EDAX spectrometer. The acceleration voltage was 15-20 kV, and the pressure 60 Pa. The samples were not coated with any conductive layer SEM and FTIR analysis were performed for Cd-contaminated soil supplemented with MCC in three selected soil:MCC ratios, 1:1, 3:1 and 7:1. Since low concentrations of Cd are undetectable by neither of these methods, soils with much higher final Cd concentrations of 50, 500, 5000 and 10000 mg/kg were used in these experiments.

### 2.5 Plants cultivation experimental setup

As a model of crop plants that could be potentially exposed to Cd phytotoxicity effect, root parsley (*Petroselinum crispum*) was used. The species is representative of plants with edible hypocotyl parts which are particularly exposed to contaminants (Dghaim et al., 2015; Lizarazo et al., 2020). Furthermore, due to the direct surrounding of potential contaminants in soil, nutrients uptake by roots and relatively low translocation factor, the concentration of contaminants in underground parts of plants is usually higher than in their aboveground parts (Zare et al., 2018; Parihar et al., 2021).

Plants were cultivated in Cd-contaminated soil (2 mg kg^-1^) enriched with MCC in a ratio 7:1 (soil:MCC). The ratio was selected based on the results of the experiment described in Section 2.3. As control variants, the soils uncontaminated and/or unsupplemented with MCC were used. Plant cultivations were performed in small pots containing 200 g of soil each. All 4 variants of the experiment (1- soil with plants, 2 - soil with MCC and plants, 3 – Cd-contaminated soil with plants, 4 - Cd-contaminated soil with MCC and plants) were carried out in 5 replicates. In every pot, five plants were grown (25 plants per experimental variant). The experiment was carried out for 14 weeks. Plant cultivation was deliberately prolonged in order to investigate the chemical stability of cadmium carbonate and observe the eventual secondary mobilization of Cd as a result of root secretion and/or soil microbiota activity. The soil and plant samples were collected at the beginning of the experiment, after 7 and/or 14 weeks.

During the plants’ cultivation experiment (i) dry mass of roots and aboveground parts of plants, (ii) water content on both parts of plants, (iii) total chlorophyll concentration as well as (iv) the total Cd concentration in plants and soil were measured. Furthermore, the analysis of soil including the pH, Cd bioavailability, quantity and activity of microorganisms was performed. Total chlorophyll concentration in plant leaves was determined *in vivo* using CI-710s SpectraVue Leaf Spectrometer (CID Bio-Science, USA) with compatible software. Spectrophotometric measurements were performed after 7 and 14 weeks of the experiment on all living leaves, with 5 single measurements per leaf.

To estimate the efficiency of cadmium uptake by plants from the soil, the bioconcentration factor (BCF) was determined. These values were calculated based on the values of total Cd concentration in soil and plant according to the equation (1):


(1) $$\text{BCF}=\frac{total\;Cd\;concentration\;in\;roots\;and\;green\;parts\;of\;plants}{total\;Cd\;concentration\;in\;soil}$$

### 2.6 Cadmium bioavailability in soil and total cadmium concentration analysis

The bioavailability of Cd in soil was determined by extraction using 100 mM CaCl_2_ solution (Chen et al., 2018). To 1 g of the soil 5 ml of calcium chloride was added and then, samples were vigorously mixed and incubated for 4 hours. Then, soil samples were centrifuged (8000 rpm, 5 minutes) and the supernatant was filtered through laboratory paper filters (Whatman 41 Grade, 150 mm diameter). The filtered samples of the soil extracts were stabilized by the addition of 69% nitric acid in a volume ratio of 4:1 and stored at a 4°C prior to the analysis.

The total Cd concentration in soil extracts, soils and plants (roots and green parts) samples was determined with the use of Atomic Absorption Spectrometry with Graphite Furnace (GF-AAS) method applying Thermo Fisher ICE 3300 spectrometer. The digestion of soil and plant samples (about 0.3 g of soil and 0.1 g of plants) was carried out with 5 ml of a mixture of 69% HNO_3_ and 30% H_2_O_2_ (4.5:0.5 v/w ratio) at 180°C for 30 min in a closed microwave system (Milestone Ethos Plus). Digested samples were transferred to plastic tubes and stored below 4°C prior to measurements. Cadmium standard solutions (Merck, Darmstadt, Germany) were prepared in 3% HNO_3_.

### 2.7 Statistical analysis

Statistical analysis of the results was performed using RStudio 2022.02.2 software. The significance of the differences between experimental groups was statistically evaluated by one-way analysis of variance (ANOVA) at p ≤ 0.05 and was represented by an asterisk under the respective group (*** - p< 0.001, ** - p ≤ 0.01, * - p ≤ 0.05, no asterisk - p>0.05). The pairwise significance of the differences between experimental variants was assessed by Tukey’s Honestly Significant Difference (HSD) test at p ≤ 0.05 and indicated by letters above the bars. The results were presented on graphs obtained with ggplot2 v3.3.5 (Wickham, 2016).

## 3 Results

### 3.1 Carbonate productivity of *Ochrobactrum* sp. POC9

**Figure 1:**
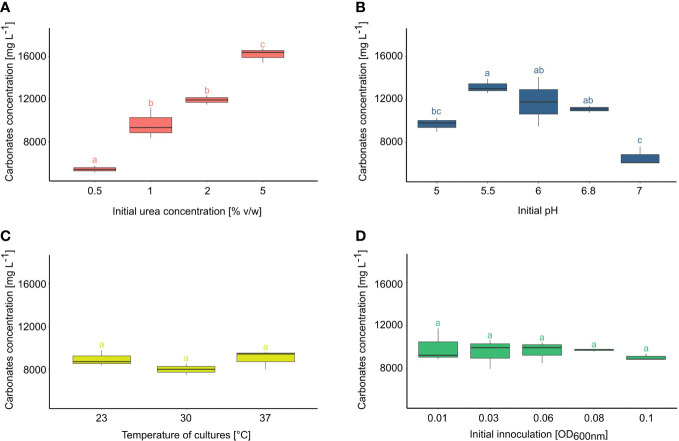
Carbonate productivity of *Ochrobactrum* sp. POC9 under the influence of various initial urea concentrations **(A)**, pH **(B)**, temperature **(C)**, and initial quantity of microorganisms **(D)**. Statistical analysis: Tukey HSD test (letters above the bars, p ≤ 0.05).

At first, the productivity of carbonate ions depending on the initial amount of urea was evaluated (**Figure 1A**). It has been noticed that the urea concentration in the medium increased the amount of CO_3_^2-^ in practically direct proportion. The highest amount of carbonates was produced with the addition of 5% urea (av.16168,33 mg/l), followed by the variant with 2% urea (av. 12371 mg/l). Next in order were variants with 1% urea (av. 9641 mg/l), and the least with 0,5% urea (av. 5458 mg/l). The most divergent (between replicates) results occurred in the version with 1% urea.

The effect of initial pH on carbonate production was also investigated (**Figure 1B**). It has been noted that out of the 5 investigated variants, three in the middle showed the highest carbonate concentrations. The highest concentration of carbonate occurred at pH 5.5 (av. 12638 mg/l) and 6 (av. 11827 mg/l). However, these results were less reproducible in comparison to the variants with an initial pH of 6.8 (av. 11135 mg/l). The result of the variant with the most alkaline pH was almost two times lower (av. 6645 mg/l) than the previous one.

No significant differences in carbonate productivity were observed among the variants at various temperatures (**Figure 1C**). The lowest concentration of carbonates occurred in cultures incubated at 30°C (av. 8010 mg/l). In the variant with incubation at room temperature slightly higher productivity (av. 8959 mg/l) than the previous one was found. Although the highest carbonate productivity was detected in microbial cultures incubated at 37 °C (av. 9018 mg/l), the observed differences were statistically insignificant.

The effect of the initial inoculation ratio on carbonate production efficiency was also evaluated (**Figure 1D**). Changes in initial optical density, related to the quantity of microorganisms, did not show notable differences in carbonate contents in microbial cultures. A variant with an initial OD_600nm_ 0.01 occurred as the one with the most uneven results. The repeatability improved with higher initial OD values. From initial OD_600nm_ 0.08, carbonate productivity seemed to be lower.

### 3.2 FTIR and SEM-EDS analysis of Cd compounds in the soil

**Figure 2:**
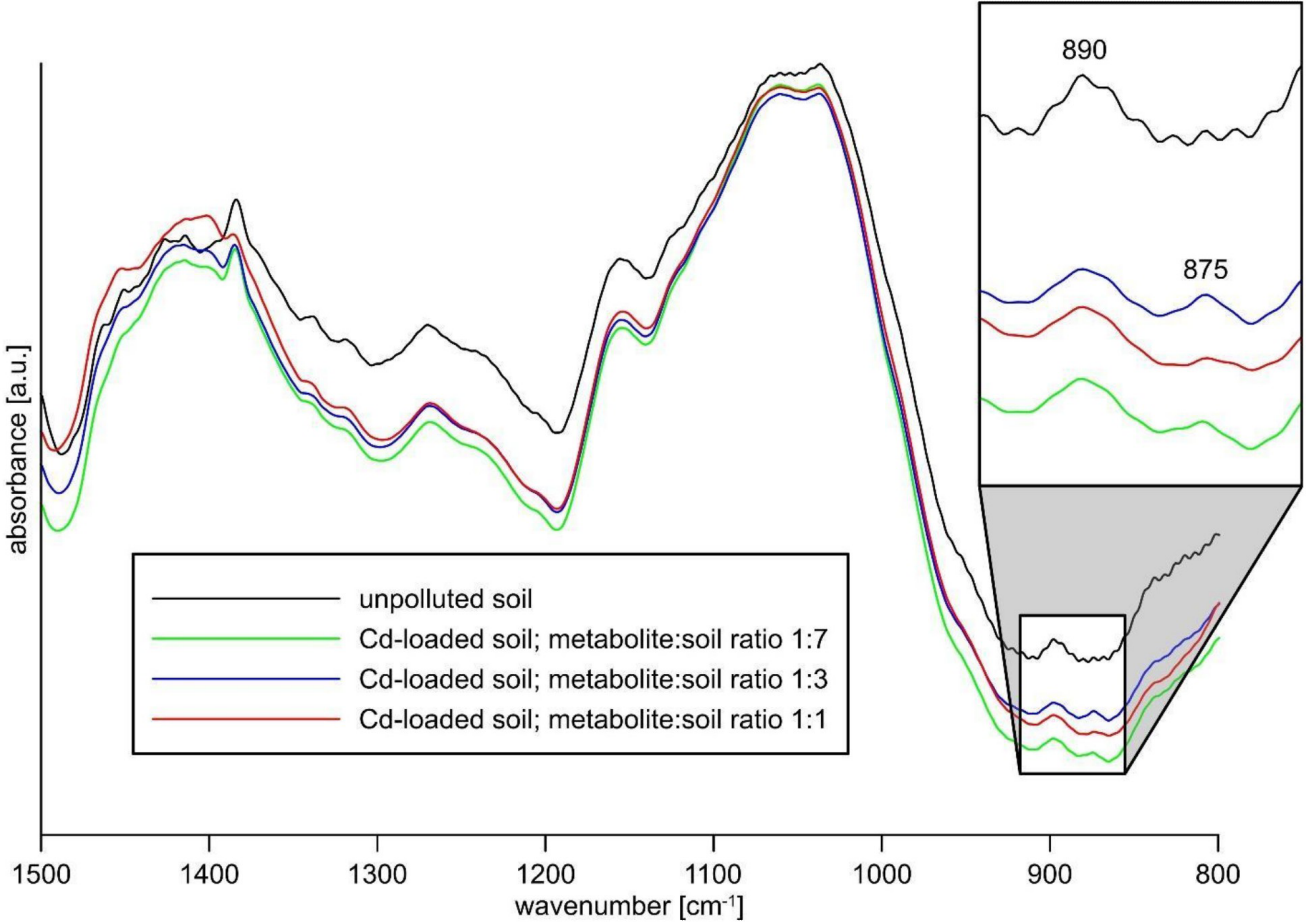
FTIR spectra of uncontaminated and Cd-contaminated (10,000 mg/kg) soil.

FTIR spectra of contaminated soil at Cd concentrations of 50 and 500 mg/kg did not virtually differ from the spectrum of non-contaminated soil. Therefore, additional analyzes of Cd-contaminated soils with cadmium at concentrations of 10,000 mg/kg were performed. Even at these high cadmium levels all the spectra were very similar (**Figure 2**). However, the spectra of Cd-contaminated soil revealed an additional weak band at ca. 875 cm^-1^ which was not observed in uncontaminated soil spectrum. The feature could be attributed to out of plane bending vibrations ν_2_ of carbonate (CO_3_^2-^) group, proving the presence of (secondary) carbonate in the soil.

**Figure 3:**
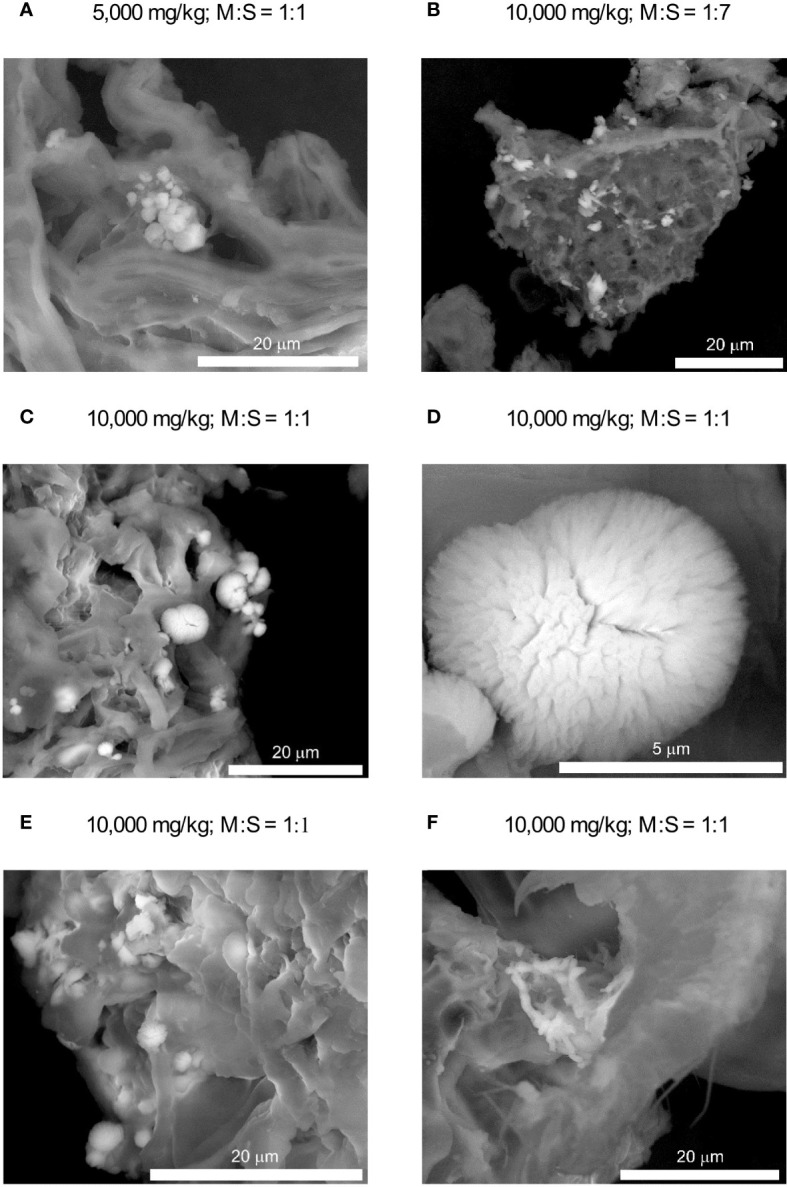
SEM images of inorganic Cd carriers in the soil (brighter crystallites). **(A, B)** – low-Cd carbonates; **(C–E)** – high-Cd carbonates, **(F)** – phosphates. M:S denotes metabolite–soil ratio.

As with FTIR analysis, the SEM observations did not reveal the presence of any separate cadmium compounds in the soils contaminated with Cd at concentrations of 50 and 500 mg/kg. Again, additional observations and analyzes were then carried out on highly contaminated soils (5000 and 10,000 mg/kg). It has been found that cadmium was apparently dispersed within organic matter, but was also incorporated, in significantly variable amounts, in inorganic compounds. Among the latter, calcium carbonate seemed to be the most common. At lower cadmium loads and/or at lower MCC/soil ratios small (up to a few micrometers in size) crystals of almost pure calcium carbonate have been encountered (**Figures 3A, B**). They showed only traces of Cd since the Cd/Ca molar ratios calculated on the basis of semi-quantitative EDS analyses are usually below 0.02–0.03. In the soil contaminated with Cd at the highest level (i.e. 10,000 mg/kg), especially at higher MCC/soil ratios, another type of carbonate accumulation has been found as well (**Figures 3C–E**). These consisted of very small, often elongated crystallites forming isometric, quasispherical or fan-like aggregates with a diameter not exceeding approximately 5–6 micrometers. Much higher concentrations of cadmium have been found in their chemical composition, as the Cd/Ca molar ratios range between 0.2 and 0.8. They usually also contain phosphate admixtures. Another inorganic cadmium carriers were apparently apatite-type calcium phosphates (**Figure 3F**). Cadmium concentrations in the phosphates are, however, rather low – Cd/Ca molar ratios did not usually exceed 0.05.

### 3.3 The effect of MCC on the pH and Cd mobility in soil

**Figure 4:**
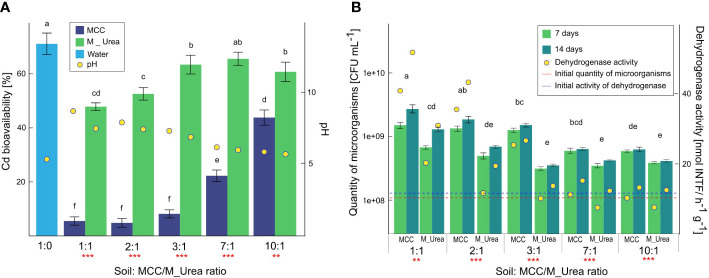
Cadmium bioavailability in soil correlated with the pH **(A)** and microbiological quantity and activity in soil **(B)** and under the influence of Cd-contaminated soil supplementation with various volumes of MCC or M_UREA after 7 and 14 days of soil incubation. Statistical analysis: ANOVA (*** - p< 0.001, ** - p ≤ 0.01) and Tukey HSD test (letters above the bars, p ≤ 0.05).

The soil supplementation with MCC contributed to the significant reduction of the Cd mobility (**Figure 4A**). The higher volume of MCC supplemented to the soil, the higher degree of Cd immobilization was observed. The most effective Cd immobilization was shown for the highest soil supplementation with MCC, in a ratio of 1:1. The level of Cd mobility in this experimental variant decreased by about 65% with regard to the control variant (0:1). A similar degree of Cd immobilization in soil was observed in the cases of ratios 2:1 and 3:1. For the 7:1 and 10:1 ratios, Cd mobility was reduced by about 51% and 29%, respectively compared to the control variant. Cadmium mobility reduction was also observed in variants with soil supplementation with urea medium used for bacteria cultivation. Furthermore, in these experimental variants, a similar correlation was found as in soil supplemented with MCC- the higher volume of medium the higher degree of Cd immobilization. In the soil supplemented with bacterial medium, the efficiency of Cd immobilization was significantly lower compared with soil enriched with MCC, regardless the soil:medium/MCC ratio. The highest Cd mobility reduction in soil supplemented with the microbial medium was found in ratios 1:1 and 2:1 (ca. 22% and 19%, respectively, compared to the control variant). For ratios 3:1, 7:1 and 10:1 the level of Cd mobility was similar and decreased by about 6-10% compared to the control variant.

The degree of Cd immobilization was strongly correlated with the pH of the soil solution resulting from its supplementation with MCC or microbial medium. It was shown that the higher the volume of MCC or medium in soil, the higher pH of the soil solution. In the case of the highest soil supplementation with MCC, the pH value increased from 5.30 to 8.54. For ratios 2:1, 3:1, and 7:1 pH values increased up to 7.76, 7.18, and 6.05, respectively. In the case of a ratio 10:1, the change in the pH value was insignificant and was 5.73. A stronger soil alkalization was observed in the soil enriched with MCC than in soil supplemented with the microbial medium. In soils supplemented with bacterial medium, the highest pH values were found in the case of a ratio 1:1, which was 7.34. The amplitudes of pH values in other soil:medium ratios were significantly lower with regard to soil supplementation with MCC and the pH in the ratio 10:1 was only slightly higher than in control soil.

### 3.4 Changes in quantity and activity of microbes in Cd-contaminated soil under the influence of MCC

The soil supplementation with MCC strongly affected the quantity and activity of soil microbiota (**Figure 4B**). Soil supplementation with MCC or bacterial medium (control variant) in various proportions contributed to the increase of the quantity of microorganisms. Furthermore, the increase in the quantity of microorganisms in the soil was directly proportional to the volumes of supplemented liquids (MCC or microbial medium). In the case of soil supplementation with MCC in the highest volume (1:1) the quantity of microorganisms increased by about an order of magnitude after 7 days of the experiment with regard to the beginning quantity (marked as a red line in **Figure 4B**). After 14 days the value increased almost twice with regard to the result obtained after 7 days, that the final quantity of microbes was 2.77*10^9^ CFU/ml. Similar correlations in the cases of 2:1 and 3:1 were observed. A lower volume of MCC also stimulated the quantity of microorganisms in the soil but the increase was lower and after 14 days of the experiment the quantities of microbes were 6.51*10^8^ CFU/ml and 3.43*10^8^ CFU/ml for 7:1 and 10:1 ratios, respectively. Thus, it was shown that even the lowest soil supplementation with MCC increased the quantity of microorganisms almost three times with regard to unsupplemented soil. The stimulation of the microbes quantity was also observed in the case of soil enrichment with bacterial medium with various volumes corresponding with MCC supplementation. Although the increase of the soil microorganisms quantity was noticeable in cases of both types of soil supplementation, the degree of biostimulation in soil enriched with the microbial medium was lower than in the case of soil supplemented with MCC by about 23-53%, depending on the soil:medium ratio.

The quantity of microorganisms directly reflected the level of the activity of microbes in the soil. This correlation was confirmed by the analysis of dehydrogenase activity in soil (**Figure 4B**). The highest activity of dehydrogenase was shown in soil supplemented with MCC in the highest volume (1:1). After 7 days of the experiment, this value was quadrupled and after 14 days increased fivefold with regard to the initial activity of dehydrogenase in unsupplemented soil. In cases of lower soil:MCC ratios, the increase of dehydrogenase activity was also significant but this decreased with the lower volumes of MCC supplemented to the soil. It was also shown that the longer soil incubation with MCC the more intensive stimulation of the activity of microorganisms. On the other hand, the soil enrichment with microbial medium with urea also contributed to the increase in dehydrogenase activity. The degree of the biostimulation of microbial activity also depended on the volume of microbial medium supplemented to the soil, but the efficiency was significantly lower compared to soil supplementation with MCC, regardless of the ratio. In cases 7:1 and 10:1, values of dehydrogenase activity in soil enriched with microbial medium after 7 days of the experiment were even below the initial value of dehydrogenase in unsupplemented soil.

### 3.5 Plants reaction to the soil supplementation with MCC

**Figure 5:**
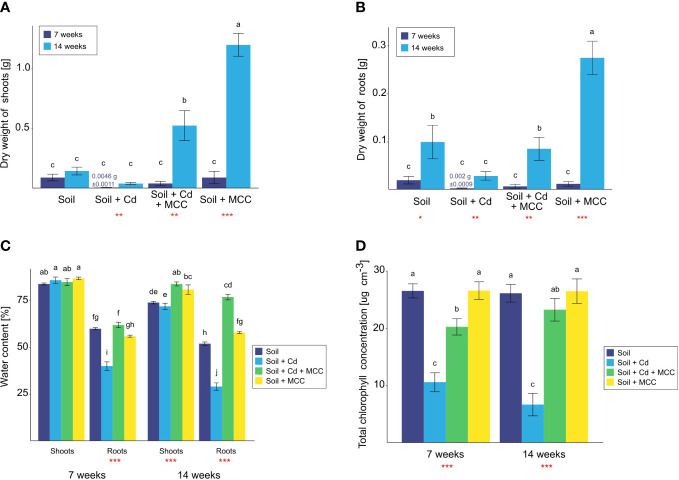
Biomass of shoots **(A)** and roots **(B)**, water content **(C)** and chlorophyll concentration **(D)** of plants cultivated in soil (un)contaminated with Cd and/or (un)supplemented with MCC after 7 and 14 days of the experiment. Statistical analysis: ANOVA (***p< 0.001, **p ≤ 0.01, *p ≤ 0.05, no asterisk - p>0.05) and Tukey HSD test (letters above the bars, p ≤ 0.05).

Soil supplementation with MCC strongly promoted plant growth and increased the biomass of both shoots and roots (**Figures 5A, B**), regardless of the Cd presence in soil. Furthermore, plant growth stimulation by the soil supplementation with MCC increased with time so the highest effect of MCC was noted after 14 weeks of the experiment. Uncontaminated soil enrichment with MCC increased the shoots biomass more than eight times with regard to shoots of plants cultivated in unsupplemented soil (**Figure 5A**). This effect was noticed after 14 weeks of the experiment – after 7 weeks of the experiment differences in shoots biomass were not observed. A similar effect of MCC was found in the case of shoots of plants grown in Cd-contaminated soil. Despite the pronounced phytotoxic effect of cadmium on plant biomass, the soil supplementation with MCC contributed to increasing shoots biomass and their dry weight was almost 15 times higher than in contaminated but unsupplemented soil. The soil enrichment in MCC also caused root growth stimulation (**Figure 5B**). The biomass of roots of plants cultivated in uncontaminated soil after 14 weeks of the experiment increased triple under the influence of MCC soil supplementation. In the case of the roots of plants grown in soil contaminated with Cd, their dry weight increased fourfold. Similarly, as in the case of shoots biomass, these differences were not observed before the end of the experiment.

Morphological changes of plants were confirmed by their physiological reaction in the form of a reduction of the water content in tissues (**Figure 5C**) and chlorophyll concentration in shoots (**Figure 5D**). Analysis of water content in plant tissues confirmed the phytotoxic effect of cadmium (**Figure 5C**). Although the turgor of shoots of plants cultivated in Cd surrounding was not changed before the end of the experiment, the strong reduction of water content in roots after just 7 weeks was observed. The soil supplementation with MCC strongly affected the general condition of plants which was confirmed by the significantly higher water content in roots already after 7 weeks of cultivation and shoots after 14 weeks of the experiment. After 14 weeks of plant cultivation, the effect of MCC enrichment was more visible since the positive influence of bacterial metabolites on the water content was observed both in the case of shoots and roots of plants grown both in pure and Cd-contaminated soil. In the case of plants cultivated in Cd-contaminated soil, the MCC contributed to the increase of turgor shoots and roots by about 12% and 48%, respectively. Furthermore, the turgor of plant roots grown in contaminated and supplemented soil was higher even than plants cultivated in pure soil. Thus, the high improvement of the general condition of plants under the influence of soil supplementation with MCC was shown.

Regarding the effect of soil supplementation with MCC on the chlorophyll concentration in plants, the positive influence was found only in the case of plants cultivated in Cd-contaminated soil (**Figure 5D**). The presence of MCC in soil contributed to an increase in chlorophyll concentration both after 7 and 14 weeks of the experiment. In the middle of the experiment, the difference in the value was twice but at the end of the experiment, there was already a fourfold difference. The effect of MCC enrichment on plants grown in uncontaminated soil was not confirmed.

### 3.6 The effect of the MCC on the Cd uptake by plants, Cd bioavailability in the soil as well as quantity and activity of soil microbes

**Figure 6:**
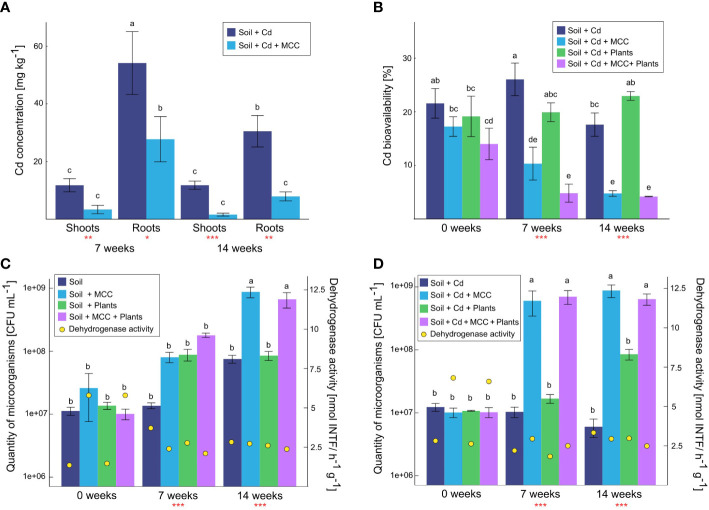
Total Cd concentration in plants cultivated in soil (un)supplemented with MCC **(A)**, cadmium bioavailability in soil **(B)**, microbiological quantity and activity in uncontaminated **(C)** and Cd-contaminated soil **(D)** after 7 and 14 weeks of plants cultivation. Statistical analysis: ANOVA (**p< 0.001, **p ≤ 0.01, *p ≤ 0.05, no asterisk - p>0.05) and Tukey HSD test (letters above the bars, p ≤ 0.05).

The soil enrichment with MCC strongly inhibited Cd uptake by plants (**Figure 6A**). Significantly lower concentrations of Cd were detected both in the roots and shoots of plants cultivated in Cd-contaminated soil, both after 7 and 14 weeks of the experiment. After 7 weeks of plant cultivation, soil supplementation with MCC contributed to the decrease of Cd concentration in shoots more than threefold while in roots the difference was twice. At the end of the experiment, the effect of soil enrichment with MCC was stronger and the differences between shoots and roots were more than sevenfold and almost fourfold, respectively, in favor of plants grown in MCC-enriched soil.

Based on the total Cd concentration in soil and plants the values of the bioconcentration factor (BCF) were calculated. BCF for plants cultivated for 7 weeks in MCC-enriched soil and in soil without MCC supplementation were 15.54 and 32.96, respectively. After 14 weeks of cultivation, BF for plants cultivated in both types of soil were lower with regard to values obtained in the middle of the experiment and were 4.74 and 21.14, respectively.

The lower Cd uptake by plants cultivated in MCC-supplemented soil was strongly correlated with the bioavailability of Cd in soil (**Figure 6B**). It has been shown that the soil enrichment with MCC significantly decreased Cd mobility both in soil without plants and in the presence of plants. These differences were visible from the beginning of the experiment, but they became more significant after 7 and 14 weeks of plant cultivation. After 7 weeks of the experiment, the difference in Cd bioavailability in soil without plants was more than twice but after 14 weeks was already almost threefold in favor of the MCC-supplemented soil. In the case of soil with plants a similar correlation was observed but differences between MCC-enriched and unenriched soil were even more significant. They were fourfold and almost fivefold, respectively. On the other hand, it was also noticed, that after 14 weeks of the experiment, the presence of plants contributed to the increase of the Cd bioavailability in the soil. In this case, the appropriate soil supplementation with MCC also reduced the cadmium bioavailability.

MCC supplementation of soil significantly affected the quantity and activity of microbes inhabiting both Cd-contaminated (**Figure 6C**) and uncontaminated soil (**Figure 6D**) regardless of the presence of plants. Furthermore, the effect of MCC on Cd-contaminated soil seemed to be stronger with regard to pure soil. In uncontaminated soil after 7 weeks of the experiment, the MCC supplementation increased the quantity of microorganisms only in soil with plants, but after 14 weeks, the effect of MCC was noticeable both in soil with and without plants (**Figure 6C**). At the end of the experiment, MCC supplementation contributed to the increase of microorganisms quantity in both types of soil by about one order of magnitude. In the case of Cd-contaminated soil, the significant effect of MCC was observed already after 7 weeks of the experiment, both in soil with and without plants (**Figure 6D**). Soil supplementation with MCC contributed in both cases to an increase in the quantity of microorganisms by almost two orders of magnitudes. After 14 weeks of the experiment, similar correlations were observed, however in this case the impact of the plants themselves on the microorganisms’ quantity was also significantly noticeable. It has been shown that the presence of plants in soil unsupplemented with MCC increased the quantity of microbes in the soil by about one order of magnitude, however, the soil enrichment with MCC increased the value by an additional one order of magnitude.

Regarding the activity of dehydrogenases, a similar effect of MCC was found both in Cd-contaminated and uncontaminated soil only at the beginning of the experiment. The activity of dehydrogenases was then higher in both types of soil under the supplementation of MCC. After 7 weeks of the experiment, significant differences in contaminated and uncontaminated soil were found. Higher activity of dehydrogenases in soil uncontaminated and unsupplemented with MCC was found, but in the case of contaminated soil, the opposite tendency was observed. Thus, in this sampling point, the positive effect of MCC supplementation was confirmed only for Cd-contaminated soil. After 14 weeks of the experiment, the positive effect of MCC supplementation in contaminated soil had been nullified, thus no effect of soil supplementation with MCC on the dehydrogenase activity in soil was found.

## 4 Discussion

### 4.1 Carbonate productivity by *Ochrobactrum* sp. POC9

As a result of urea hydrolysis in presence of urease, ammonium and carbonate ions are created (Zambelli et al., 2011) that directly affect cadmium mobility in soil. Ammonium ions increase the pH of soil, contributing to the reduction of cadmium mobility whereas carbonate enables the precipitation of insoluble cadmium carbonate (Li et al., 2013). Changing conditions of the urea hydrolysis reaction affect the activity of urease, which corresponds directly to the content of both types of products in the solution (Krajewska, 2009). In our study, a positive correlation between the amount of urea and the concentration of carbonate in the medium was observed. This phenomenon is an effect of the Michaelis–Menten equation, which indicates that at a constant enzyme content, the efficiency of the reaction depends, within certain limits, on the substrate concentration (Krajewska, 2016). A similar correlation was found in other ureolytic bacteria strains NB33, LPB21, NB28, NB30 belonging to *Sporosarcina pasteurii* and *Bacillus lentus*. In these cases, the lowest urease activity at 2% of urea concentration, and highest at 6 or 8% initial urea concentrations was detected (Omoregie et al., 2017). With 10% of the initial urea concentration, the amount of hydrolyzed urea decreased, probably by the inhibition of the ureolysis reaction by the products (Krajewska, 2016). The carbonate production efficiency of the *Ochrobactrum* POC9 strain was maximally 16168.33 mg/l when the medium contained 5% of urea. The carbonate efficiency was a bit higher than in other previously described strains, e.g., *Bacillus pasteuri* (Yi et al., 2021).

For the MICP process, the change in the initial pH of the microbial medium can affect the activity of urease, and through it the amount of carbonate ions (Barazorda-Ccahuana et al., 2020). We showed that pH range 5.5- 6.8 is optimal for *Ochrobactrum* sp. POC9 growth. Previous studies of urease enzyme kinetics confirmed that ureolysis is a pH dependent process (Fidaleo et al., 2003; Lauchnor et al., 2015). Furthermore, the optimum pH for cell-free extract was narrower than this in the presence of bacteria and was more vulnerable to changes in pH to acidic values (van Paassen, 2009). This was one of the reasons why cell-free supernatant was used in our study. The activity of urease may vary in various bacteria species depending on the pH (Sachs et al., 2001). For *Pararhodobacter* sp. maximum urease activity was found at pH 8 (Fujita et al., 2017). For *Bacillus pasteurii* ATCC11859 optimum pH range was 7.0-9.0 (Yi et al., 2021) and for *Sporosarcina pasteurii* optimal is pH 9.25, and when pH raised to 10, the growth of bacteria was stopped (Wiley and Stokes, 1962). *Micrococcus yunnanensis* showed the highest urease activity on pH 7 (Imran et al., 2019). It is regarded that urease activity is significantly lower in acidic conditions (lower than 5) and alkaline (over 8), which was also confirmed in our study.

No significant differences in carbonate production efficiency were observed between various temperature variants. A few other experimental results showed that the temperature was positively correlated with the ureolysis rate (van Paassen, 2009; Krajewska, 2016) and microbial cultures incubated at the higher temperature (e.g., 37 °C) gave the best results. Differences in response to this factor can be seen between *Sporosarcina pasteurii* and *Bacillus megaterium* (Sun et al., 2019). The urease activity of *B. megaterium* was lower in temperatures between 25 and 30 °C but in the case of *S. pasteurii*, the activity of the enzyme increased with the temperature. For *Bacillus pasteurii* optimal temperature was 30 °C (Yi et al., 2021). Furthermore, in this temperature, the highest urease activity has been noticed.

Changes in initial optical density, related to the quantity of microorganisms, did not show notable differences in carbonate contents in POC9 cultures. The most probable explanation is the difference in bacterial growth rate and the relative abundance of nutrients available for the bacterial population. In variants with a higher quantity of bacteria, nutrient availability was lower than in variants with a less initial quantity of microbes (Allen and Waclaw, 2019). In other studies, an initial optical density of cultures strongly affected the urease activity, eg. in *Pararhodobacter* sp. culture (Fujita et al., 2017). Urease activity increased almost in directly proportional to the increase of bacterial optical density variants. For *Sporosarcina pasteurii* (DSM 33) the effect of three initial optical densities (OD_600nm_ 0.2, 1.0, 3.0) on the growth and urease activity was tested (Wang et al., 2021). It has been shown that in the variant with the lowest initial OD_600nm_ bacteria growth was the most intensive. The highest examined variant had the lowest bacterial growth rate and the fastest entered the lethal phase. This result was explained again by shortages in nutrients.

### 4.2 Cadmium carbonate detection in the soil by SEM and FTIR analysis

The presence of a carbonate mineral in the Cd-contaminated soil is evidenced by the emergence of an additional feature at ca. 875 cm^-1^ in the FTIR spectra (Russell, 1977; Bucca et al., 2009; Liu et al., 2017; Pérez-Villarejo et al., 2018). Due to a very low intensity and significant blurring of this band, it is not possible to precisely determine its position and consequently to determine which carbonate is present. The approximate position at ca. 875 cm^-1^ is, however, close to those reported for both calcium (calcite 876–878 cm^-1^, vaterite 877 cm^-1^) and cadmium (otavite 860–863 cm^-1^) carbonates (Jones and Jackson, 1993; Russell and Fraser, 1994; Moreno et al., 2018; Liu and Lian, 2019). Although this value is closer to calcium carbonates, the clear asymmetry of the band (**Figure 2**) might suggest the crystallization of a CaCO_3_-CdCO_3_ solid solution as well. Such a possibility is favored by the close similarity of calcium and cadmium divalent cations, especially by small difference between Cd^2+^ and Ca^2+^ ionic radii which are 0.95 Å and 0.99 Å, respectively (Shannon, 1976; Tesoriero and Pankow, 1996). Therefore complete miscibility of (Ca,Cd)CO_3_ has been evidenced experimentally (Sackey et al., 2019; Mergelsberg et al., 2021). Since the soil contains much more calcium than cadmium, the supply of carbonate to the system results in the crystallization of larger amounts of calcium carbonate. However, due to the clear difference in the solubility products (K_SP_) of both compounds (10^-8.48^ for CaCO_3_ vs 10^-12.24^ for CdCO_3_), it can be assumed that cadmium will be preferentially incorporated into precipitating calcium carbonate. The formation of (Ca,Cd)CO_3_ solid solution at the surface of calcium carbonate crystals is also possible via Ostwald ripening during recrystallization processes (Kang et al., 2014a).

Scanning electron microscopic observations confirmed crystallization of carbonate minerals on the surface of Cd-contaminated soil (**Figure 3**). Lower Cd concentrations in the soil and/or at lower soil:MCC ratios resulted in the formation of small blocky or platy crystals with recognizable rhombohedral habits and a low Cd admixture (**Figures 3A, B**). Such a morphology, combined with the FTIR feature at ca. 875 cm^-1^ (**Figure 2**), suggests that the carbonate is calcite. This is consistent with previous studies since calcite is apparently the most common product of MICP (Kumari et al., 2014; Krajewska, 2018). Higher Cd levels and higher soil:MCC ratios favored crystallization of (Ca,Cd)CO_3_ that revealed other habits - acicular or columnar crystals that form isometric or fan-like aggregates (**Figures 3C–E**). The morphology combined with EDS chemical analyses suggest calcite-otavite solid solution. A distinct change in morphology of Cd-substituted Ca carbonates was observed previously (e.g. Kim et al., 2021; Natsi and Koutsoukos, 2022) and resulted probably from a change in crystallization kinetics. Both Ca and Ca-Cd carbonates are apparently more common in samples of higher soil:MCC ratios. This is more likely due to the alkalizing effect of metabolites on the soil solution (**Figure 4A**), which in turn favors the precipitation of carbonates.

### 4.3 The efficiency of Cd immobilization in soil with the use of MICP

The reduction of Cd mobility in soil with the use of MICP method is caused by the increase of pH in soil and the precipitation of cadmium carbonate (Lyu et al., 2022). Due to the high-efficiency Cd immobilization even in low concentration in soil, recently MICP is more and more frequently used for the treatment of soil dedicated to agricultural purposes (Liao et al., 2023). In our study, the MICP was used for Cd immobilization in agricultural soil, and furthermore, the soil bioconsolidation method based on MICP was optimized not only in the context of the carbonate production maximization but also regarding the soil supplementation manner.

The results of Cd bioavailability in soil reflected an increased degree of immobilization of this element under the influence of soil supplementation with MCC produced by the POC9 strain. Application of MCC to Cd-contaminated soil contributed to the significant reduction of the Cd bioavailability; however, the efficiency of the process strongly depended on the soil:MCC volume ratio. Reduction of the Cd bioavailability ranged from 28 to 65% depending on the volume of MCC supplemented to the soil. The positive effect of MICP in regard to Cd immobilization was confirmed also for many other bacterial strains belonged i.e. to *Bacillus* (Zhao et al., 2017; Wang et al., 2019b), *Lysinibacillus* (Kang et al., 2014b), *Pseudomonas* (Bravo et al., 2018), *Serratia* (Bhattacharya et al., 2018), and *Sporosarcina* (Kang et al., 2014a) genus, but those studies concerned the Cd precipitation in various solutions/media, not in soil. In solutions, the possibility of Cd precipitation with carbonates is usually easier and more effective than in soil since the higher mobility of both types of molecules in a liquid matrix (Yang et al., 2016; Ghorbanzadeh et al., 2020). Regarding the soil experiments, the obtained Cd precipitation with the use of MCC produced by the POC9 strain was comparable with other previously described bacteria as eg. *Enterobacter* spp. (56.10%) (Peng et al., 2020), *Sporosarcina pasteurii* (61.1-89.3% (Ghorbanzadeh et al., 2020) or 13.6%- 29.9% (Liu et al., 2021), and soil autochthonous microbiota (59.8%) (Lyu et al., 2022).

The positive effect of Cd-contaminated soil supplementation with medium containing urea without MCC was also found. In these experimental variants, the effect of biostimulation of autochthonous microbiota by the additional nutrition was observed. It has been shown that soil enrichment with 2% of urea stimulated the ureolytic activity of autochthonous soil microorganisms, which contributed to increased Cd immobilization. In our study, the efficiency of this process was significantly lower with regard to soil supplementation with MCC but still noticeable. Immobilization of Cd and other heavy metals by MICP based on the activity on the autochthonous soil microbiota was previously described (Kim and Lee, 2019; Lyu et al., 2022) and the efficiency of the process were comparable with data obtained in this study.

Most of the so far described MICP applications for heavy metal immobilization in soil utilized culture breeding (bacterial cells suspended in metabolites solution) directly to contaminated soil (Kumari et al., 2014; Liu et al., 2021; Qiao et al., 2021). In some cases, soil enrichment with the exogenous pool of microbes may have a negative effect on the soil’s autochthonous microbiome condition, by disturbing its natural community structure (Lourenço et al., 2018; Seitz et al., 2022). The appropriate microbiome composition is particularly important in the context of the treatment of agricultural soil since microbes directly interact with crop plants and affect their productivity (Trivedi et al., 2020; Glick and Gamalero, 2021). Furthermore, the unfavorable effect may be enhanced by autochthonous and exogenous microbes competition about space and/or nutrients (Mawarda et al., 2022; Nunes et al., 2022), which may also affect the MICP efficiency (Liu et al., 2019). In our study, a filtered MCC solution was applied to Cd-contaminated soil, thus minimization of the interference of allochthonous microorganisms in the soil ecosystem was ensured.

### 4.4 Biostimulation of soil microbiota by MCC

The addition of external carbon sources is essential for the proper growth and activity of soil microbes (Bandara et al., 2021). Application of various organic amendments to soil results in the improvement of bacterial community structure, diversity and quantity (Omori et al., 2016; Macias-Benitez et al., 2020; Tao et al., 2020; Readyhough et al., 2021). The addition of MCC to soil was correlated with a significant increase in microorganism quantity, observed in soil contaminated with Cd and uncontaminated. Soil supplementation with MCC provided an additional nutrient pool, which could stimulate bacterial growth. As was shown in our previous studies, various secondary metabolites were produced during the decomposition of urea by *Ochrobactrum* sp. POC9 (Yang et al., 2021). Among them, unsaturated hydrocarbons were one of the major groups. These compounds could be used by various ecological groups of bacteria as energy sources (Widdel and Musat, 2016). Secondary metabolites of *Ochrobactrum* sp. POC9 also contained a high amount of organic acids, which could be an easily-accessible carbon and energy source for microorganisms (Macias-Benitez et al., 2020). It was also shown that organic acids play a crucial role in the stimulation and recruitment of plant-growth-promoting rhizobacteria, being one of the major components of plant roots exudates (Macias-Benitez et al., 2020; Sharma et al., 2020). Alcohols were another major group of organic compounds found in secondary metabolites of *Ochrobactrum* sp. POC9. Bacteria with various lifestyles are able to degrade alcohols (Fisk et al., 2009; Yakushi and Matsushita, 2010) and use them as an energy source (Yakushi and Matsushita, 2010; Schuchmann and Müller, 2016).

The increase in the quantity of microorganisms was also observed in soil contaminated with Cd due to decreased bioavailability of this heavy metal. High concentrations of Cd have toxic effects on bacterial cells, possibly causing DNA damage, disturbed cell division, inhibition of respiration and enzymatic activity, mainly through binding to sulfhydryl groups in proteins (Trevors et al., 1986; Fazeli et al., 2011). The presence of extensive Cd in soil has also impacted entire microbial communities, reshaping their structure and activity resulting in decreased bacterial biomass and diversity (Pal et al., 2005; Yu et al., 2021). The addition of MCC to soil was correlated with a decrease in Cd bioavailability, which is a crucial factor for its toxicity (Bandara et al., 2021). This created more optimal conditions for microbial communities to grow and resulted

in greater microbial quantities. In other papers, it has been shown that the reduction of Cd bioavailability improved soil microbiome in terms of quality and quantity (Bandara et al., 2021; Sun et al., 2021). An increase in microbial basal respiration and biomass was observed in soils, as an effect of the addition of biochar, which decreased Cd bioavailability (Bandara et al., 2021). Stabilization of Cd could also have an indirect positive impact on soil microbiota in the plant rhizosphere. Reduced toxicity of Cd for plants results in their stimulated growth. This elevates the level of soil plants residues and root exudates, providing nutrients for various soil microbes (Sun et al., 2021).

In contrast to the observed stimulation of the number of microorganisms in the presence of plants, a decrease was observed in dehydrogenase activity (DHA). DHA play a key role in the oxidation of organic matter mediating the transfer of hydrogen from the substrates to acceptors. DHA is involved in the transfer of hydrogen atoms to coenzymes such as NAD ^+^, NADP ^+^ or FAD ^+^, which are the first acceptors of the electrons in cellular respiration (Furtak and Gajda, 2017). Therefore, it is considered that the activity of dehydrogenase is indirectly connected with soil microorganisms quantity (Skujins and McLaren, 1967). The activity of DHA in soil depends on many factors, including pH, soil hydration level, redox potential, oxygen diffusion rate, organic matter content, or temperature. In this work, it was shown that despite the increase in the quantity of microorganisms in the soil, the activity of the DHA decreased during the experiment. In several studies, the optimal soil pH for DHA activity was neutral or slightly alkaline and was in a range of 7-8.5 (Włodarczyk et al., 2002; Ros et al., 2003). Moreover, low activity of DHA has been observed below pH 6.6 and above pH 9.5 (Trevors, 1984). These correlations may explain the decrease in DHA activity during experiments, despite the increase in the quantity of microorganisms. After the initial addition of MCC to the soil, its pH increased providing optimal conditions for the activity of DHA. In the course of the experiment, a decrease in the soil pH was observed, resulting in suboptimal conditions and lowering the DHA activity.

### 4.5 Effect of MCC on the general plants’ conditions and Cd uptake efficiency

Cadmium contamination of soil negatively affects seed germination and the synthesis of photosynthetic pigments, reduces crop plant growth and number of leaves as well as inhibits root elongation (Mani et al., 2012; He et al., 2017; el Rasafi et al., 2020). Cadmium is regarded as highly toxic and its accumulation in plant tissues results in disturbance of various intracellular processes. Cadmium stress induces the overproduction of reactive oxygen species (ROS), which eventually leads to damage and destruction of cell membranes, organelles and biomolecules (Abbas et al., 2018). In this study, Cd presence in the soil also caused a significant inhibition of crop plants development, observed already after 7 weeks of cultivation, compared to plants grown in soil supplemented with MCC. The presence of MCC had a highly positive effect on the overall condition of plants. The biomass of plants grown in soil without MCC was significantly lower and leaves discoloration was observed, in contrast to the plants grown in soil with MCC, which remained in good condition throughout the experiment. A similar correlation was observed for the plants grown in unpolluted soils with and without the supplementation, but this effect was found after 14 weeks of cultivation. The deterioration of the condition of plants at the end of the experiment could have been the result of a shortage of micro- and macroelements in the soil (Kumar et al., 2021). Due to the addition of MCC, the plants were supplied with the necessary building components until the end of the cultivation, so their development was not inhibited (Styczynski et al., 2022).

Supplementation of MCC to soil provided also an additional source of nitrogen, which is a crucial element for plant growth and development (Xu et al., 2012; Kiba and Krapp, 2016; Feng et al., 2020). Ammonium ions are directly introduced with MCC to soil, where they are present as a product of urea hydrolysis (Zambelli et al., 2011). Ammonium is considered as a preferred nitrogen source for plants, because it does not have to undergo further transformation after uptake inside of the plant cells (Xu et al., 2012; Kiba and Krapp, 2016). This direct utilization of ammonium by plant limits energy expenditure on nitrogen assimilation (Hachiya and Sakakibara, 2017). However, in several studies it was shown that uptake of nitrate is also important for plant growth, despite the necessity of its reduction to ammonium either in roots or leaves (Hachiya and Sakakibara, 2017; Feng et al., 2020). MCC could also indirectly contribute to the supply of nitrate in soil available for plant uptake. Soil is inhabited by nitrifying bacteria, which are able to oxidize ammonium delivered with MCC to nitrite and nitrate (Beeckman et al., 2018; Wendeborn, 2020). MCC also creates preferential conditions for oxidation, by alkalization of soil. Neutral to slightly alkaline soil pH is correlated with highest efficiency of soil nitrification (Norton and Stark, 2011).

The results regarding water and chlorophyll content indicated a positive effect of MCC supplementation. The presence of MCC allowed for maintaining constant hydration of the plants and photosynthetic activity throughout the cultivation period. This could be connected with mitigation of Cd toxicity in plants. It has been shown that exposure to Cd in soil could lead to osmotic stress and lowering of water content in shoots (Rizwan et al., 2016; Haider et al., 2021). Cadmium also affects chlorophyll content, mainly by hindering the uptake and metabolism of sulfur and iron, which are crucial for chlorophyll biosynthesis (Gallego et al., 2012; Haider et al., 2021). The beneficial effect of a representative of the genus *Ochrobactrum* on plant growth was also described in cucumber culture supplementation with *Ochrobactrum* sp. NW-3. Furthermore, NW-3 has been classified as plant growth-promoting bacteria (PGPB) (Xu et al., 2015). Other examples of PGPB strains may be *Bacillus* sp. UR21, which contributed to the increase in the biomass and chlorophyll content in pakchoi plants cultivated in Cd-contaminated soil (Wei et al., 2022) or *Pseudomonas grimontii* Bc09*, Pantoea vagans* So23*, Pseudomonas veronii* E02, and *Pseudomonas fluorescens* Oj24 that had a positive effect on acetic acid production, 1-aminocyclopropane-1-carboxylic acid deaminase (ACCD) activity and phosphate solubilization by *Panicum virgatum L.* cultivated in Cd-contaminated soil (Begum et al., 2018).

In this study, we showed that most of the Cd ions absorbed by plants were retained in the root system. The reason for this is structural elements of the cell wall, including pectin compounds and proteins, protect plants against contaminant translocation (el Rasafi et al., 2020). MCC soil supplementation resulted in a strong inhibition of Cd uptake by plants and significantly lowered accumulation of Cd in the tissues, both in the root and shoot parts. Literature data also indicate a positive effect of the use of ureolytic bacteria *Bacillus* sp. UR21 and eggshell waste on the reduction of Cd uptake by pakchoi plants (*Brassica chinensis* L.) (Wei et al., 2022). A similar correlation was also confirmed in the case of *Bacillus* sp. TZ5, which effectively increased the biomass of ryegrass and decreased Cd accumulation in those plants (Ma et al., 2020) or in the case of *Pantoea agglomerans*, which promoted rice growth and reduced Cd concentration in rice grain (Tian et al., 2022).

## 5 Conclusions

The soil supplementation with MCC produced by *Ochrobactrum* sp. POC9 as a result of the hydrolysis reaction contributed to the significant reduction of Cd bioavailability in soil and improved its microbiological properties by the increase in the quantity of soil microorganisms. Both cadmium immobilization in the form of carbonates, as well as additional nutrition of soil by its enrichment with MCC, resulted in the better growth of plants and a significant reduction in Cd uptake. It has been shown that appropriate soil supplementation with MCC produced by the POC9 strain may bring a quadruple beneficial effect in the form of Cd toxicity reduction, stimulation of the soil microbes growth, reduction in the Cd uptake by plants, and promotion of plants’ growth. Furthermore, the soil supplementation with MCC did not negatively affect the properties of the soil and thus this method may be successfully applied in soil dedicated to agricultural purposes.

## Data availability statement

The raw data supporting the conclusions of this article will be made available by the authors, without undue reservation.

## Author contributions

Conceptualization: KD-A. Data curation: MZ, GR, MM, AG, RS. Formal analysis: KD-A. Funding acquisition: KD-A, GR. Investigation: MZ, GR, MM, AG, RS. Methodology: MZ, GR, KD-A. Project administration, resources and supervision: KD-A. Validation: MZ, GR, MM, AG, RS, KD-A. Visualization: GR, MM, KD-A. Writing—original draft: MZ, GR, MM, AG, KD-A. Writing—review & editing: MZ, GR, MM, RS, KD-A. All authors contributed to the article and approved the submitted version.
