# Supplementary material for: Reduction of bioavailability and phytotoxicity effect of cadmium in soil by microbial-induced carbonate precipitation using metabolites of ureolytic bacterium Ochrobactrum sp. POC9
Source: Front Plant Sci. 2023 Jun 21;14:1109467. doi: 10.3389/fpls.2023.1109467 (PMC10321601; doi:10.3389/fpls.2023.1109467)
Supplement: Supplementary file 1 [file DataSheet_1.zip › Supplementary information.pdf]

Supplementary materials:

Investigation of the mechanism of cadmium precipitation in the solution with the use of MCC

Methodology

The MCC was obtained according to the procedure described in the main manuscript - section 2.1. In an additional experimental variant, the POC9 strain was cultivated in an M-UREA medium avoiding the source of phosphorus. In both variants, the beginning pH of the medium was adjusted to 6.8. After 72 hours of incubation, the bacterial culture was centrifuged (3000 rpm, 15 minutes). The obtained supernatants were added to $CdCl_2x2H_2O$ solution with a cadmium content of 100 mg/L in an amount of 12.5% (v/v). The MCC-cadmium mixtures were incubated for 24 hours with shaking at 150 rpm and then centrifuged (10 000 rpm, 15 minutes). The obtained precipitates were dried at 60 °C and then were characterized using FTIR, SEM-EDS and powder X-ray diffractometry PXRD. X-ray diffraction patterns were collected using Rigaku SmartLab instrument equipped with a graphite monochromator, rotation Cu anode, 45 kV and 200 mA generator settings. Analyzes were performed in 2–75°2Θ recording range with 0.05° step size, and a counting time of 1 second per step. The XRD patterns were evaluated using a diffraction pattern database of the International Centre for Diffraction Data.

Results and discussion

These analyses indicate that a reaction of MCC with Cd solution results in the formation of a compact precipitate consisting chiefly of amorphous cadmium phosphate (Fig. SM1). The lack of crystallinity is evidenced by the XRD pattern showing only two very broad diffraction bands at approximately 2.9 and 1.8 Å (Fig. SM2), resembling those reported earlier for amorphous Cd phosphates (Matusik et al., 2008; AbuShanab et al., 2020). In the FTIR spectrum of the precipitate (Fig. SM3) two intense absorption bands coming from P-O and O-P-O vibrations are visible at ca. 1013 cm^{-1} and ca. 570 cm^{-1}, which can be attributed to asymmetric stretching vibrations (ν_3) and asymmetric bending vibrations (ν_4), respectively (Matusik et al., 2008; Wołowiec et al., 2019). The broad feature at ca. 1647 cm^{-1} arises probably from adsorbed molecular water (Russell & Fraser, 1994). A cadmium carbonate admixture and/or the presence of carbonate anion in cadmium phosphate structure is proved by infrared absorption bands at 1459, 1391 and 853 cm^{-1} (Fig. SM3) attributable to CO_3 asymmetric stretching (ν_3) and out of plane bending (ν_2), respectively (van der Marel & Beutelspacher, 1976). On the other hand, the reaction of MCC with Cd in the absence of phosphate results in the precipitation of crystalline cadmium carbonate (otavite) as proven by both X-ray diffraction pattern (Fig. SM2) and FTIR spectrum (Fig. SM3) of the precipitate. The latter shows typical $CdCO_3$ infrared features at ca. 721 cm^{-1} (ν_4), ca. 860 cm^{-1} (ν_2), ca. 1073 cm^{-1} (ν_1), ca. 1413 cm^{-1} (ν_3), and ca. 1790 cm^{-1} ($\nu_1 + \nu_4$) (Askarinejad & Morsali, 2008; Bucca et al., 2009; Moreno-Morales et al., 2018). Otavite forms uniformly distributed small (up to ca. 1–2 μm) two-piece 'snowman-like' particles made by attached roundish crystallites (Fig. SM1) or – less often – individual oval particles below 1 μm. The presence of a large amount of small crystallites indicates a strong supersaturation of the solution.

Figure captions

Fig. SM1. SEM images of the solids obtained by reaction of MCC with cadmium in the presence (left) and absence (right) of phosphate.

Fig. SM2. PXRD diffraction patterns of the solids obtained by reaction of MCC with cadmium in the presence (blue) and absence (red) of phosphate. The numbers above peaks denote d_{hkl} distances in Å. All the peaks visible in the red pattern are attributable to otavite ($CdCO_3$, ICDD card number 42-1342).

Fig SM3. FTIR spectra of the solids obtained by reaction of MCC with cadmium in the presence (blue) and absence (red) of phosphate.

References

AbuShanab W.S., Moustafa E.B., Hammad A.H., 2020. Dependence of the structure, optical, and dynamic properties of novel cadmium phosphate glass on vanadium content. Journal of Materials Research and Technology 9, 14179–14189.

Askarinejad A., Morsali A., 2008. Syntheses and characterization of $CdCO_3$ and CdO nanoparticles by using a sonochemical method. Materials Letters 62, 478–482.

Bucca, M., Dietzel M., Tang, J., Leis A., Köhler, S.J. (2009). Nucleation and crystallization of otavite, witherite, calcite, strontianite, hydrozincite, and hydrocerussite by CO_2 membrane diffusion technique. Chemical Geology, 266, 143–156.

Matusik J., Bajda T., Manecki M., 2008. Immobilization of aqueous cadmium by addition of phosphates. Journal of Hazardous Materials 152, 1332–1339.

Morales M.G.E., Garcia A.M.E., Cruz C.S., Plata B.R., Moreno P.O., Pérez G.R., 2018. $CdCO_3$ nanocrystalline thin film grown by chemical bath and its transition to porous CdO by thermal annealing treatment. Optik 171, 347–355.

Russell J.D., Fraser A.R., 1994. Infrared methods. In: Wilson M.J. (Ed.) Clay Mineralogy: Spectroscopic and Chemical Determinative Methods, Chapman & Hall, London, 11–67.

Van der Marel H.W., Beutelspacher H., 1976. Atlas of Infrared Spectroscopy of Clay Minerals and Their Admixtures. Elsevier, Amsterdam.

Wołowiec M., Tuchowska M., Kudła P., Bajda T., 2019. Synthesis and characterization of cadmium chlorapatite $Cd_5(PO_4)_3Cl$. Mineralogia 50, 3–12.
