# Supplementary figures and images for: Reduction of bioavailability and phytotoxicity effect of cadmium in soil by microbial-induced carbonate precipitation using metabolites of ureolytic bacterium Ochrobactrum sp. POC9
Source: Front Plant Sci. 2023 Jun 21;14:1109467. doi: 10.3389/fpls.2023.1109467 (PMC10321601; doi:10.3389/fpls.2023.1109467)

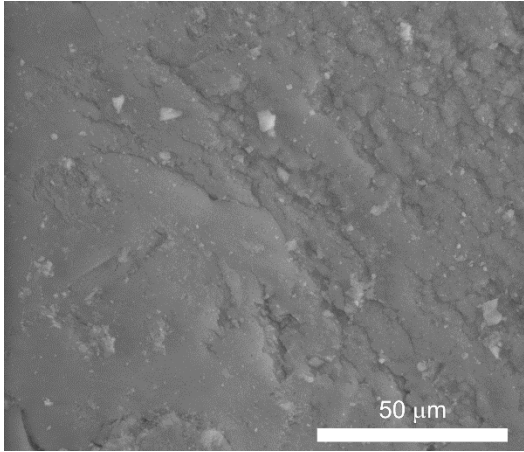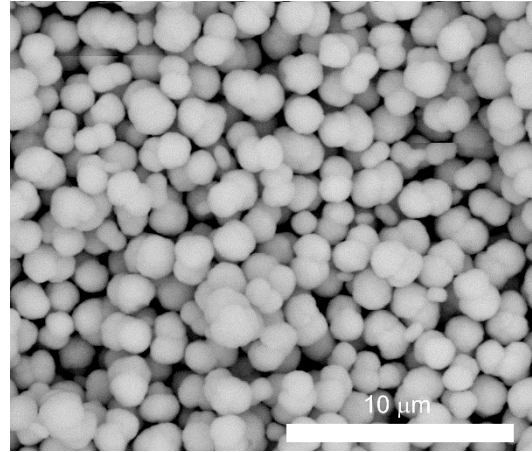

Supplement: Supplementary file 1 [file DataSheet_1.zip › Fig. SM1.pdf]

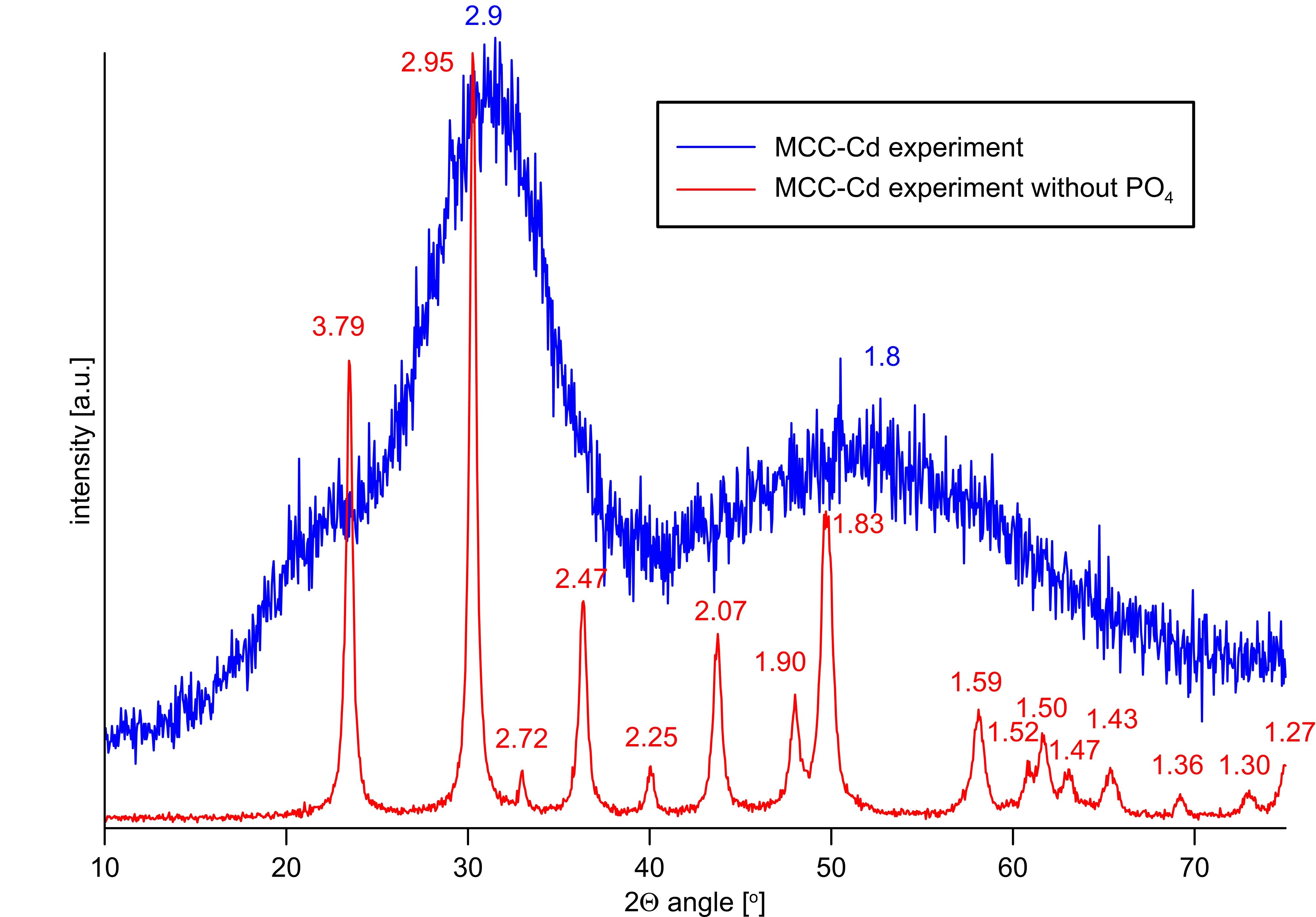

Supplement: Supplementary file 1 [file DataSheet_1.zip › Fig. SM2.jpeg]

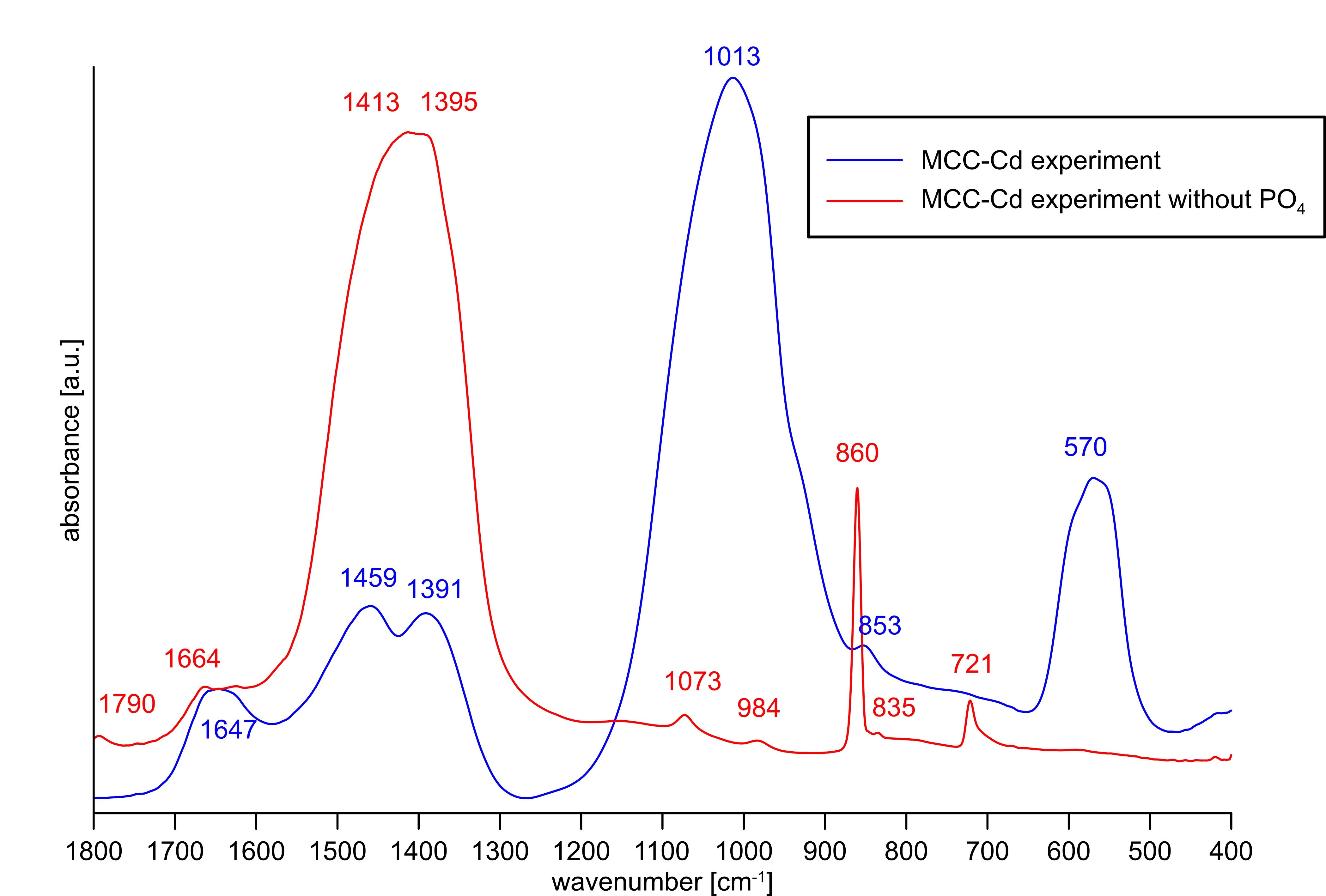

Supplement: Supplementary file 1 [file DataSheet_1.zip › Fig. SM3.jpeg]
